# Editorial: Horizon 2030: Innovative Applications of Heart Rate Variability

**DOI:** 10.3389/fnins.2022.937086

**Published:** 2022-06-03

**Authors:** Sylvain Laborde, Emma Mosley, Clint Bellenger, Julian Thayer

**Affiliations:** ^1^Department of Performance Psychology, Institute of Psychology, German Sport University Cologne, Cologne, Germany; ^2^Normandie Université Caen, Unité de Formation et de Recherche des Sciences et Techniques des Activités Physiques et Sportives, Caen, France; ^3^Solent University, Southampton, United Kingdom; ^4^Allied Health and Human Performance Unit, Alliance for Research in Exercise, Nutrition and Activity (ARENA), University of South Australia, Adelaide, SA, Australia; ^5^South Australian Sports Institute, Adelaide, SA, Australia; ^6^Department of Psychological Science, University of California, Irvine, Irvine, CA, United States

**Keywords:** heart rate variability (HRV), parasympathetic nervous system (PNS), vagus nerve, vagus nerve (VN) stimulation, wearable

## Introduction

**Figure 1:**
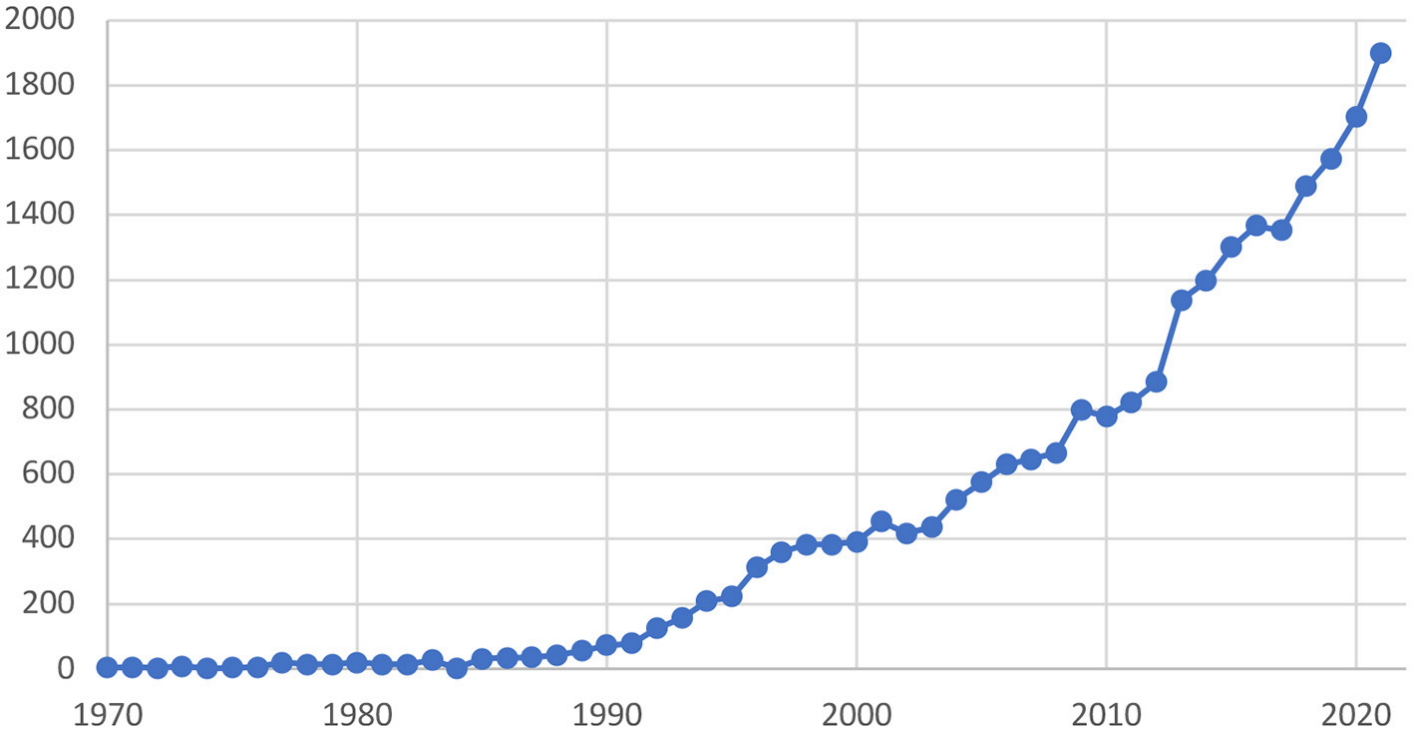
Yearly frequency of publications mentioning “heart rate variability” in PubMed.

The Guest Editors are delighted to showcase 39 papers for this Frontiers Research Topic (RT) “Horizon 2030: Innovative Applications of Heart Rate Variability”. We are thankful to all 139 contributors, representing institutions from 16 countries (See Table 1). There is a growing genuine interest in this topic as there have been over 175,000 views as of April 2022. This is also a reflection of the growing use of heart rate variability (HRV) in scientific publications. The number of studies mentioning HRV since the 1970s has grown significantly and is now reaching almost 2000 papers per year (see Figure 1). This rapid growth makes it challenging to see and understand the broader interpretation of research findings. This Editorial provides a contextual overview to our RT, building upon previous influential Frontiers RT on HRV (e.g., Billman et al., 2015, 2019; Drury et al., 2019), with a view to highlight conceptual considerations for HRV in the future.

**Table 1:** List of countries represented in the Research Topic, based on first author's institution.

**1st author institution country**	**Number**
United States	11
Italy	9
Germany	5
Netherlands	2
United Kingdom	2
Australia	1
Austria	1
Canada	1
China	1
France	1
Israel	1
Japan	1
New Zealand	1
South Korea	1
Spain	1

*A 16^th^ country was also represented within the affiliation of co-authors, Switzerland*.

Prior to discussing the contributions, the Guest Editors will put this RT into a broader context, anchoring it within classical work, and showcasing how it can advance current HRV knowledge. HRV, the variation in the time intervals between adjacent heartbeats (Malik, 1996; Berntson et al., 1997; Laborde et al., 2017), has become one of the most popular psychophysiological measures in recent times. Its use by researchers and practitioners spans across many different fields, encompassing medicine, the health sciences, psychology, exercise and sport sciences, and other disciplines. The growing popularity of HRV is perhaps due to its desirable characteristics: non-invasive, low cost, and its recording feasibility across a large range of settings. Above all, HRV is capable of indexing the activity of the vagus nerve - the main nerve of the parasympathetic nervous system - regulating cardiac functioning, through cardiac vagal activity (also referred to as cardiac vagal tone) (Malik, 1996; Berntson et al., 1997; Laborde et al., 2017). Cardiac vagal activity is associated with many functions in self-regulation, adaptation, and health (Thayer and Lane, 2009; Thayer et al., 2009; Shaffer et al., 2014; Laborde et al., 2017, 2018b; Smith et al., 2017), thus making it a key marker of interest to researchers.

In order to guide readers through the broad diversity of the papers constituting this RT, we organize and integrate the contributions in relation to theoretical, methodological, and applied considerations related to HRV.

## HRV Theory: the Need to Base One's Research on Solid Theoretical Foundations

One of the primary goals of this RT was to address an aspect often neglected in HRV research, namely the theoretical foundations supporting it. Given the accessibility of HRV measurement, it is tempting to use it for any application researchers may deem interesting. However, given the dozens of HRV parameters that one can calculate based on RR interval data (for an overview of HRV parameters and their physiological underpinning, see Laborde et al., 2017, Table 1), this therefore increases the likelihood of significant statistical relationships when all variables are explored. Consequently, to interpret collected data, and to understand phenomena associated with HRV, we need theories. Researchers often highlight the importance for more theoretically driven work and developing overarching theoretical frameworks in order to develop research hypotheses and draw comprehensive conclusions (Muthukrishna and Henrich, 2019). As a metaphor, instead of randomly accumulating bricks on a building site, theories allow us to build the foundations and structure of a house.

The main influential theories in HRV research have been reviewed in previous summary works (Shaffer et al., 2014; Laborde et al., 2017), and the most comprehensive theoretical perspective to date has been the Neurovisceral Integration Model (NIM; Thayer et al., 2009; Smith et al., 2017). In the present RT, 60% of papers use the NIM framework. Importantly, all theories related to HRV have a core component: the ability of HRV to index cardiac vagal activity, which enables the understanding of the role of HRV in phenomena related to self-regulation (Holzman and Bridgett, 2017).

A recent additional consideration to the NIM was the Vagal Tank Theory (Laborde et al., 2018b), which stresses the need to go beyond only considering resting HRV levels, to also take into account the reactivity to the task/event, and the recovery from the task/event, to achieve a comprehensive understanding of cardiac vagal activity as indexed by HRV. In the present RT, HRV reactivity was investigated by Condy et al., who found different associations for HRV at rest and HRV reactivity with the outcomes investigated; and by Borges et al., who controlled for resting HRV to interpret the relationship between reactivity HRV, transcutaneous vagus nerve stimulation (tVNS), and cognitive functioning.

After considering which theoretical approach is adequate to provide a solid understanding background for their research questions, researchers need to pay close attention to HRV methodological guidelines, given the influence methodological choices may have on HRV data interpretation.

## Methods: the Importance of Following HRV Methodological Guidelines

Methodological aspects have to be considered when conducting HRV research, in order to ensure correct interpretation of the data (Laborde et al., 2017). A range of papers in this RT contribute to methodological development in HRV research. As we mentioned above, HRV theories focus on the ability of HRV to index cardiac vagal activity, and we will now refer to these parameters as vagally-mediated HRV (vmHRV). Examples of parameters reflecting vmHRV are the root mean square of successive differences (RMSSD), or high-frequency HRV when the respiratory frequency is comprised between 9 and 24 cycles per minute (Malik, 1996; Berntson et al., 1997; Laborde et al., 2017).

A common query surrounds the duration of HRV measurement. Seminal work of the HRV Task Force (Malik, 1996) pointed toward the standardized use of both short-term (5 min) and long-term (24 h) measurements in order to allow comparison across research labs. However, some phenomena may not necessarily fit those durations and researchers have started to examine shorter measurements opportunities. Within this RT, Shaffer et al. performed a critical review of ultra-short-term (UST) HRV measurements (≤ 5 min). The overarching message from this paper was that the validity of UST measurements remains questionable at this point in time. Specifically, the research reviewed lacked rigorous investigation of criterion validity (which indicates that a novel measurement procedure produces comparable results to a currently validated measurement tool). Another recurring issue is the correction of artifacts, given a single false heartbeat can dramatically alter HRV metrics (Berntson and Stowell, 1998). Those challenges remain to be addressed before a widespread use of UST HRV measurements can be recommended.

Kim et al. further investigated the validity of UST HRV in non-static conditions, which would provide greater application in activities of daily life. However, they found that longer measurements are needed in dynamic conditions in comparison to static conditions to obtain reliable results. It is important to note that dynamic conditions also give rise to interpretational challenges, since metabolic influences on HRV have to be taken into account in this context.

This question was investigated by Brown et al., while assessing six methods to optimally detect episodes of non-metabolic vmHRV reduction as an indicator of psychological stress in everyday life. They found that a 24 h measurement detected the largest percentage of episodes of reduced additional vmHRV that matched self-reported stress levels. While this method was the most promising, using the first 10 min from three consecutive hours was also a good indicator. Another illustration of the use of 24 h measurement is found in Jarczok et al., where the authors describe how 24 h HRV measurements can be used as a communication tool for a personalized psychosomatic consultation in occupational health.

The devices used to measure HRV can also influence the interpretation of HRV data. While electrocardiogram (ECG) measurement remains the gold standard (Laborde et al., 2017), chest belts (e.g., Bellenger et al.) and photoplethysmography measurements (Mejia-Mejia et al.) enable HRV measurements in a broader range of contexts. Mejia-Mejia et al. investigated pulse-rate variability, which refers to HRV information obtained from pulse wave signals such as photoplethysmography measurements. They found that pulse-rate variability may quantify different, more localized information about the autonomic nervous system in the periphery of the body, which was thought to be due to the location of the pulse-rate variability monitor when compared to more central HRV measurements.

In the consideration of HRV parameters, we recommend researchers select them in line with theorical underpinning of the research. Researchers should also be mindful of the purported physiological mechanisms they are suggested to reflect. While editing the papers submitted to this RT, we noticed many studies still rely on the low-frequency/high-frequency (LF/HF) ratio, and the so-called autonomic or sympathovagal balance. This interpretation was originally based on the premise that LF provided an index of sympathetic nervous activity, a claim that has now long been disproved in the literature, in particular using blockade studies (for a recent overview, see Ackermann et al., 2021).

While HRV alone cannot provide a full overview of the autonomic nervous system, its pairing with impedance cardiography for this purpose seems promising. Indeed, impedance cardiography enables assessment of the preejection period (PEP, the time elapsed between the electrical depolarization of the left ventricle and the beginning of ventricular ejection), which has been recognized as the gold standard index of sympathetic nervous activity (Sherwood et al., 1990). In the present RT, Wiley et al. provided evidence for the use of the left-ventricular ejection time (LVET) instead of PEP for the same purpose. Like HRV, LVET is indeed a measure of chronotropic influence (reflecting the control of the heart via the sinoatrial node), while PEP is a measure of inotropic influence (reflecting myocardial contractility).

One way to further develop HRV theory is to investigate HRV together with other psychophysiological measurement techniques, such as with functional near-infrared spectroscopy (fNRIS), as performed by Condy et al.. This combined methodological approach based on neuroimaging allows for direct testing of the NIM. More specifically, this enables the investigation of pre-frontal activation via oxygenated hemoglobin to provide a critical test of the assumptions of the NIM, with vmHRV, which helps illustrate how task demands and environmental contexts have to be taken into account for accurate interpretation of HRV.

Finally, in addition to *in vivo* measurements, *in silico* investigation (i.e., based on computer modeling) also prove to be beneficial in answering specific research questions. For example, *in silico* investigation was used to clarify the different impact of HRV in the deep cerebral and central hemodynamics at rest (Scarsoglio et al.). One of the interesting findings was that an increase in HRV per se does not seem to be sufficient to trigger a better cerebral hemodynamic response.

After showcasing the diversity of methodological advances addressed within our RT, we now move toward the domains of applications that emerged from the studies of this RT.

## HRV Horizon 2030: Diving Into Tomorrow's HRV

The Guest Editors certainly share a juvenile enthusiasm for the fantastic opportunities and insights offered by HRV research, however, they are also among the first ones to recommend extreme care regarding the claims that are made about HRV. This position is brilliantly illustrated by Karemaker in his opinion piece. Karemaker, an experienced HRV researcher who published his first HRV paper shortly after the first Guest Editor of this Research Topic was born (DeBoer et al., 1984), provides us with a stimulating and witty reflection about exaggerated claims made with HRV: “*The heart may be a mirror of the soul, but the human mind is more than its heart rate variability*”. Karemaker uses a fictional case study taking place in 2030, illustrating the issues that may stem from a society geared toward preventive medicine. In this scenario, implanted biochips tracking HRV and other biomarkers watch over the health of the global population, with artificial intelligence analyzing the massive data flow to support the diagnostic process. Considering this insight into the future and warnings regarding over interpretation of HRV data, we now look to this peer reviewed RT to discover the exciting applications of HRV.

### HRV Across the Lifespan

HRV measurement has relevance at any age. At the beginning of the lifespan, Chiera et al. reviewed the literature to support the use of HRV monitoring in neonatal intensive care units. Specifically, they elaborate on a routine measurement integrating infants' HRV metrics, vital signs, and past history, in order to develop models capable of efficiently monitoring and predicting the infant's clinical conditions, enabling healthcare to improve in every stage of the perinatal period (from conception to the first years of life).

At the other end of the lifespan continuum, Hernandez-Vicente et al. focused on extreme longevity. They showed a reduction of vmHRV (and other HRV parameters) with age, which could be representative of a natural exhaustion of allostatic systems related to age. HRV may thus be considered an indicator of healthy aging.

### Psychology

HRV proves to be useful in the investigation of many psychophysiological phenomena, specifically when considering vmHRV. vmHRV was found to help further understand psychological concepts, for example compassion (Di Bello et al.). In their study, Di Bello et al. found vmHRV to be associated distinctively to compassion components, lower vmHRV for the empathic component (i.e., having the pain resonate in oneself), and higher vmHRV for the action component (i.e., engaging in actions aimed to alleviated self or others suffering), which helped to shed light on the different nature of compassion components.

Zammuto et al. investigated HRV together with the theory of mind (ToM), which is the human ability to infer the mental states of others to understand their behaviors and plan their own actions. The results preliminarily suggest that resting vmHRV might be used as an indicator of the ability to understand the content of mind of others. Furthermore, vmHRV may also be connected to morality. Lischke et al. found a positive association between individuals' vmHRV and moral rule adherence, implying that individuals with efficient integration abilities were more inclined to follow moral rules than individuals with inefficient integration abilities.

Regarding interoceptive accuracy, Lischke et al. found a positive association with vmHRV. Given the role played by our interoceptive ability to perceive and interpret changes in our autonomic nervous system, which then influence our emotional experiences, this finding is of high importance to psychological wellbeing. Furthermore, vmHRV can also be used to index the cardiorespiratory response to environmental challenges associated with specific personality characteristics. For example, Martino et al. investigated the cardiorespiratory response to moderate hypercapnia in female college students expressing behaviorally inhibited temperament. Despite baseline differences in behaviorally inhibited individuals (lower LF-HRV), no differences were found with the non-behaviorally inhibited individuals in response to hypercapnia, both groups showing an increase in vmHRV, reflecting an adaptation mechanism.

Investigating vmHRV as a potential biomarker for coping with constant discrimination, Rosati et al. investigated the cardiovascular conundrum in sexual minorities. The cardiovascular conundrum is a paradoxical profile of greater elevated sympathetic vasoconstriction (increased total peripheral resistance) and increased vmHRV, which has been reported in African Americans both at rest and in response to orthostasis. The authors found a similar pattern of response in sexual minorities, another group frequently exposed to constant discrimination.

When considering the relationship between vmHRV, depression, and positive affect, Spangler et al. found that they were influenced by gender. This study also provides guidance for researchers to look beyond linear relationships between HRV and specific outcomes, to consider also the possibility for quadratic relationships. Investigating worries and general anxiety disorder symptoms, Fishback et al. found that worriers who have higher levels of top-down control capacity (as indexed by vmHRV) may initiate and persist in worry, at least initially, because they value it.

In organizational psychology, vmHRV was suggested to be an important marker of work exhaustion, in combination with other psychological variables, such as self-esteem (De Longis et al.). Finally, Lohani et al. used HR and vmHRV to investigate arousal and cognitive demands in manual and partially automated driving, and no differences were found in drivers new to partial automation.

To summarize, the research presented in this RT showcases the diverse implementation of HRV, specifically vmHRV, in psychological research.

### Medicine

Contributions to this RT illustrate that HRV can be very useful in the medical field, as a prevention and diagnostic tool.

HRV assessment was proposed to be valuable in the assessment of autonomic dysfunction linked to the motor activity of the human colon (Ali et al.). More specifically, during propulsive motor patterns, an overall shift in autonomic activity with an increase in parasympathetic control was found.

In cardiology, HRV was found to have prognostic value after acute myocardial infarction (Hayano et al.). The authors found that mortality risk in post-acute patients with low left ventricular ejection fraction is predicted by indices reflecting decreased HRV or HR responsiveness and cardiac parasympathetic dysfunction, whereas in patients without low left ventricular ejection fraction, the risk is predicted by a combination of indices that reflect decreased HRV or HR responsiveness and an indicator that reflects large abrupt HR changes, suggesting sympathetic involvement.

Regarding temporal lobe epilepsy (TLE), resting HRV measurement was used to identify the influence of lateralization on cardiac dysfunctions (Dono et al.), showing that left TLE is associated with higher vmHRV than right TLE. Left TLE patients may consequently have a lower risk of developing cardiac dysfunctions, and hence be less susceptible to develop Sudden Death for Epilepsy.

Finally, HRV may prove useful in cancer monitoring. Wu et al. found an association between HRV and breast tumor stage, and HRV parameters may help construct an effective early diagnostic and clinical prognostic model.

To summarize, routine assessment of HRV appears beneficial to improve the prevention and diagnostic of a large range of medical conditions.

### Large-Scale Health Assessment

One of the attractive characteristics of HRV is the possibility to measure it remotely with a diversity of devices, that become more accessible to a larger audience each day, enabling integration of HRV measurements to large-scale health monitoring strategies.

The role of HRV in the future of remote digital biomarkers is considered by Owens. Specifically, remote HRV assessment has potential as an adjunct digital biomarker in neurovisceral digital phenotyping that can add continuously updated, objective and relevant data to existing clinical methodologies, aiding the evolution of current “diagnose and treat” care models to a more proactive and holistic approach that pairs established markers with advances in remote digital technology. Remote HRV assessment also enables 24 h measurements, which can then be used as a communication tool for a personalized psychosomatic consultation in occupational health (Jarczok et al.).

Greater accessibility makes HRV very important during the COVID-19 pandemic, given vmHRV can provide a sensitive measure of inflammatory processes and immunomodulation. Drury et al. provides a nice illustration of this concept with the use of the Oura ring, a ring able to monitor vmHRV continuously via PPG.

We understand that the interested reader may desire field-based HRV measurements, and we therefore provide some suggestions for further reading around additional devices based on PPG beyond the Oura ring that would also enable large scale vmHRV assessment, in particular via smartphone apps using chest belts (e.g. Polar, Garmin, Suunto), smartwatches (e.g., Apple Watch, Fitbit), or smartphone apps like Elite HRV, Kenkou, and HRV4Training (Plews et al., 2017; Altini and Plews, 2021). An overview of the accuracy of popular commercial technologies that measure resting HRV can be found in Stone et al. (2021).

In summary, we are intrigued by the possibilities offered by HRV measurements applied to a large scale, facilitated by the use of remote measurement technologies.

### Sport and Exercise

Sport and exercise science is a growing area within HRV research, given athletes and coaches have discovered the benefits of monitoring their HRV to adjust training load and optimize recovery to achieve best performance (Stanley et al., 2013; Buchheit, 2014; Bellenger et al., 2016).

HRV may help to reduce the cost of testing and training (Rogers et al.). Rogers et al. presented a new detection method based on HRV defining the aerobic threshold for endurance exercise and training prescription. This detection method may substitute existing procedures (i.e. formal gas exchange testing or invasive blood lactate sampling) which have the characteristics of being costly, requiring special test equipment, trained operators, as well as ongoing calibration and verification.

Considering the monitoring of training-induced adaptations, Bellenger et al. investigated the impact of functional overreaching on post-exercise parasympathetic reactivation in runners. Importantly, they showed increased post-exercise vmHRV following heavy training in functionally overreached athletes, which may seem paradoxical, given increases in post-exercise vmHRV are also observed in response to improvements in performance. Consequently, this study very nicely illustrates the need to consider additional measures to provide more context to vmHRV measurements, such as subjective training tolerance in the case of athletes.

Regarding the monitoring of stress-recovery status, Barrero et al. used HRV during a Cycling Grand Tour (female version of the 2017 Tour de France), with a specific test, the so-called orthostatic test (transitioning from lying down to standing up). Specifically, a low HR and vmHRV index change between supine and standing positions was found to reflect a maladaptive training stress-recovery status, with HR change having a higher predictive value than vmHRV change.

Finally, in terms of training planning, we know that individualization is crucial. Hottenrott et al. used HRV for coaching an elite endurance athlete during and after viral infection. Specifically, they showed the extent to which vmHRV and HR can be used to individualize training recommendations instead of following general rules. In a similar vein to Barrero et al., they used the orthostatic test, together with HR and vmHRV indicators, and found that this procedure was useful for detecting viral diseases early when implemented in daily routines.

In summary, HRV, and specifically vmHRV appears a promising way for athletes and coaches to monitor and help improve performance and wellbeing.

## vmHRV Enhancement

Given vmHRV is associated with a large range of positive outcomes, understanding how to enhance it is of great interest for researchers and practitioners (Fatisson et al., 2016; Laborde et al., 2018a,c).

One of the most effective methods to increase vmHRV is the voluntary slowing and pacing of one's breath, slow-paced breathing, to a frequency around 6 cycles per minute (Laborde et al., 2021, 2022; Sevoz-Couche and Laborde, 2022), a technique that has also been referred to as HRV biofeedback (Lehrer et al., 2020).

Three papers focus on slow-paced breathing in this RT which showcase noteworthy advances of the traditional paradigm. Shaffer and Meehan provide a practical guide to resonance frequency assessment for HRV biofeedback. If a respiratory frequency of 6 cycles per minute was found to be beneficial across individuals (Lehrer et al., 2020), performing slow-paced breathing at the individual resonance frequency may provide even higher benefits (see also the corrigendum to this article). Further, Patron et al. showed that the benefits of slow-paced breathing with biofeedback could be enhanced introducing competitive settings, in a sample comprising highly competitive individuals (manager). Finally, Tatschl et al. examined how implementing slow-paced breathing with biofeedback as an adjunctive therapy during inpatient psychiatric rehabilitation. They found that slow-paced breathing facilitates recovery of depressive symptoms and enhances autonomic functioning short-term, with a 1-year pre-post intervention follow-up study. This unique longitudinal study nicely illustrates how slow-paced breathing with biofeedback can be used as an adjunct, safe and non-invasive complementary treatment to help individuals with depression.

Regarding other techniques aimed at stimulating the vagus nerve, we find ostheopatic treatment, where HRV analysis is proposed to evaluate the effectiveness of osteopathic manipulative treatment as a preventive or complementary strategy in clinical and non-clinical conditions characterized by autonomic dysfunction (Carnevali et al.). Furthermore, non-invasive brain stimulation also offers options to stimulate the vagus nerve, specifically with tVNS, showing that it can increase cognitive flexibility in a set-shifting paradigm (Borges et al.). However, the mechanism in which tVNS influences vmHRV is less conclusive.

In summary, techniques aimed at increasing vmHRV are promising, given the positive outcomes linked to increased vmHRV.

## Animal Research

As a last domain of application, beyond human research, it appears meaningful to investigate HRV in animals, to develop animal models which may help to better understand HRV in humans. Piantoni et al. investigated HRV together with selective pharmacological autonomic blockades, to document an age-related impairment in cardiac vagal modulation in mice, which is consistent with the human condition. Given their short life span, mice could be further utilized as an aged model for studying the trajectory of vagal decline with advancing age using HRV measures.

Finally, Shemla et al. investigated the beating rate variability of isolated mammal (rabbit and mouse) sinoatrial node tissue, providing insight into its contribution to HRV. Different trends were found between beating rate and beat rate variability or HRV in isolated sinoatrial node tissue vs. recordings collected under *in vivo* conditions, respectively, implying a complex interaction between the sinoatrial node and the autonomic nervous system in determining HRV *in vivo*.

In summary, animal HRV models, especially developed in mammals, may provide very interesting insights that would help us to better understand HRV in humans.

## Conclusion

This RT showcased the current richness and diversity of HRV, as well as novel approaches in emerging research. Many challenges remain open at the Horizon 2030, and we are glad to have contributed to the evolution of the field with this RT, respectfully standing on the shoulders of the giants from the Task Force of the European Society of Cardiology and the North American Society of Pacing and Electrophysiology (Malik, 1996). We recognize the influence of our colleagues who started this revolution in HRV research more than three decades ago, as well as of those who relentlessly pursued these endeavors since the end of the 20^th^ century. Exciting HRV challenges are ahead of us, as illustrated by the contributions to this RT summarized in this Editorial.

Keeping in mind the warning of Karemaker expressed above, the Guest Editors would like to encourage researchers and practitioners to bravely start or continue their journey to integrate HRV to our lives. Finally, given individuals cannot solely be surmised by their HRV, we trust that HRV can provide a nice starting point toward helping making the world in which we live more ***para**sympathetic*.

## Author Contributions

SL wrote the first draft of this manuscript. EM, CB, and JT provided very useful critical feedback. All authors contributed to the article and approved the submitted version.

## Conflict of Interest

The authors declare that the research was conducted in the absence of any commercial or financial relationships that could be construed as a potential conflict of interest.

## Publisher's Note

All claims expressed in this article are solely those of the authors and do not necessarily represent those of their affiliated organizations, or those of the publisher, the editors and the reviewers. Any product that may be evaluated in this article, or claim that may be made by its manufacturer, is not guaranteed or endorsed by the publisher.
